# On the Monte Carlo weights in multiple criteria decision analysis

**DOI:** 10.1371/journal.pone.0268950

**Published:** 2022-10-07

**Authors:** Jiří Mazurek, Dominik Strzałka

**Affiliations:** 1 Department of Informatics and Mathematics, School of Business Administration in Karvina, Silesian University in Opava, Opava, Czech Republic; 2 Department of Complex Systems, Faculty of Electrical and Computer Engineering, Rzesźow University of Technology, Rzesźow, Poland; University Campus Bio-Medico of Rome, ITALY

## Abstract

In multiple-criteria decision making/aiding/analysis (MCDM/MCDA) weights of criteria constitute a crucial input for finding an optimal solution (alternative). A large number of methods were proposed for criteria weights derivation including direct ranking, point allocation, pairwise comparisons, entropy method, standard deviation method, and so on. However, the problem of correct criteria weights setting persists, especially when the number of criteria is relatively high. The aim of this paper is to approach the problem of determining criteria weights from a different perspective: we examine what weights’ values have to be for a given alternative to be ranked the best. We consider a space of all feasible weights from which a large number of weights in the form of *n*−tuples is drawn randomly via Monte Carlo method. Then, we use predefined dominance relations for comparison and ranking of alternatives, which are based on the set of generated cases. Further on, we provide the estimates for a sample size so the results could be considered robust enough. At last, but not least, we introduce the concept of *central weights* and the measure of its robustness (stability) as well as the concept of alternatives’ *multi-dominance*, and show their application to a real-world problem of the selection of the best wind turbine.

## 1 Introduction

Multiple criteria decision making/aiding/analysis (MCDM/MCDA) methods represent one of the most successful tools for sophisticated decision making in the framework of complex real-world problems usually involving many alternatives which should be compared and ranked under a set of suitable criteria. A state-of-the-art on MCDM/MCDA methods can be found e.g. in [1–3] or [4]. One of the main challenges associated with MCDM/MCDA that attracted a broad range of studies is the problem of appropriate (objective) derivation of criteria weights, see e.g. [5–15], or [16]. According to [9], the methods for the derivation of criteria weights fall into three categories–subjective weighting methods, objective weighting methods and hybrid weighting methods. Subjective methods depend on the preferences of decision-makers and include direct ranking, point allocation, pairwise comparisons, or SMART (Simple Multi-Attribute Ranking Technique). The main disadvantage of these methods is that they are recourse-consuming when the number of criteria increases. Objective weighting methods utilize specific computational process based on the initial data or decision-matrix, and are not based on experts’ preferences or judgements; entropy method, CRITIC (CRiteria Importance Through Inter-criteria Correlation) or SECA (Simultaneous Evaluation of Criteria and Alternatives) belong in this category. The hybrid weighting methods, such as MEREC (MEthod based on the Removal Effects of Criteria), combine both approaches.

In general, MCDM/MCDA methods suffer from two drawbacks related to criteria weights. The first is that it is almost impossible to set weights of criteria precisely (perfectly) so that the optimal solution is obtained indeed. This occurs, in particular, when facing a new challenge or a novel and a decision maker is lacking corresponding knowledge and/or experience, typically when cutting edge science or technology is involved. However, even relatively simple tasks constitute a problem. For instance, *QS university ranking*, which aspires to provide an ordered list of top 1,000 universities in the World as close to the reality as possible, applies the following criteria: academic reputation (40%), employer reputation (10%), faculty/student ratio (20%), citations per faculty (20%), international student and faculty ratio (both 5%). However, how do we know that these exact criteria weights provide a truly objective and unbiased ranking of universities?

The second drawback relates to the well-known high sensitivity of criteria weights with respect to the final evaluation of alternatives. In some cases even the slightest change in criteria weights may lead to a diametrically different ranking of alternatives and/or the change of the best one, see e.g. [17], and this is the case when natural uncertainties in the evaluation of criteria or alternatives arise. NASA belongs among institutions famous for their meticulous approach to the space exploration and problem solving in general. Their *NASA Systems Engineering Handbook* in the part 6.8 Decision Analysis states [18]:

Once the decision alternative evaluation is completed, recommendations should be brought back to the decision maker including an assessment of the robustness of the ranking (i.e., whether the uncertainties are such that reducing them could credibly change the ranking of the alternatives). Generally, a single alternative should be recommended. However, if the alternatives do not significantly differ, or if uncertainty reduction could credibly alter the ranking, the recommendation should include all closely ranked alternatives for a final selection by the decision-maker.

This paragraph clearly acknowledges uncertainty in the evaluation of alternatives and stresses the importance of a robustness analysis.

In this paper we turn over the usual perspective on MCDM/MCDA problems. Instead of asking which alternative is the best under given criteria weights we ask what the values of criteria weights have to be so that given alternative is ranked first. Further on, we ask what smallest change in criteria weights leads to the change of the best alternative and whether there are feasible weights such that *any* alternative could be ranked first at all.

The aim of our study is to answer these questions by proposing a novel Monte Carlo weights’ approach. We consider the set of all feasible weights (a subspace of an *n*-dimensional space, where *n* is the number of criteria) from which a large number of weights (see Section 4 for estimates of the sample size with respect to the relative standard error of the mean) in the form of *n*-tuples is drawn randomly from a uniform probability distribution via Monte Carlo method. Then, we apply predefined dominance relations for comparison and ranking of alternatives, and we provide an analysis of the sensitivity (robustness) of the aforementioned solutions by introducing a concept of the so called *central weights* and their *radius*. Moreover, we show that although an alternative can be non-dominated when compared with a single other alternative, a group domination (called *multi-domination*) may appear: an alternative might be dominated by a subset of other alternatives for all feasible weights. The identification of multi-dominated alternatives can significantly reduce the number of alternatives under consideration as demonstrated in the application part of the paper. At last, but not least, our approach accentuates the problem of uncertainty mentioned in the NASA Systems Engineering Handbook by modelling the values of criteria weights, thus allowing examination of their influence on alternatives’ final rankings.

The organization of paper is as follows: in Section 2 we provide a brief introduction to the Monte Carlo method, Monte Carlo weights and dominance relations along with an illustrative numerical example. In Section 3 we demonstrate the application of our approach to the analysis of a concrete real-world problem, namely a selection of the best wind turbine. Discussion (Section 4) and Conclusions (Section 5) close the article.

## 2 The method

### 2.1 The Monte Carlo method

In general, the term *Monte Carlo method* refers to a broad variety of algorithms that obtain numerical results via (many times) repeated random sampling from a given probability distribution. See e.g. [19–22] or [23] for an introduction to the Monte Carlo method.

History of Monte Carlo method dates back to the Buffon’s needle problem for the derivation of the value of *π* from the 18^*th*^ century. The modern version of the Monte Carlo method was pioneered during the World War II by Stanislaw Ulam and John von Neumann [22, 24]. Since then, Monte Carlo method was successfully applied in physics (see e.g. McKean–Vlasov processes), mathematics (complex multidimensional definite integrals), economics (Markov chains, risk), engineering (oil extraction), biology (study of genomes and proteins), medicine (radiotherapy), sports, or operations research (optimization problems), see e.g. [25–28], or [29]. In the context of pairwise comparisons, Monte Carlo studies were applied for instance in [30–38], or [39]. Nevertheless, a comprehensive review of Monte Carlo applications is beyond the scope of this study. In [28] Alex Bielajew states that up to year 2011, more than 300,000 papers were published on the Monte Carlo method, with 10% of papers related to medicine only.

Currently, the Monte Carlo method constitutes a popular modelling method in a wide areas of human action supported by many software products such as GoldSim, NIST, or B-RISK, and Monte Carlo simulation modules are also included in MS Excel as XLSTAT, in the statistical software SPSS, in MATLAB, or in the programming language R.

Usually, the Monte Carlo methods follow the following steps: 1) the domain of sampling and probability distribution are defined, 2) a large number of random draws (with repeating) is performed, 3) results are aggregated, analysed and interpreted.

The application of Monte Carlo method requires a random (unbiased) sampling from a given probability distribution. In practice, pseudo-random sequences are generated by a class of algorithms called *pseudorandom number generator* (*PRNG*), or a *deterministic random bit generator* (*DRBG*), see e.g. [40]. For example, MS Excel uses the *Mersenne Twister algorithm* (*MT19937*). Pseudo-random sequences are easy to test and re-run. The only quality usually necessary to make a good simulation is that a pseudo-random sequence is ‘random enough’.

The second crucial feature of the Monte Carlo simulations is their error. Each time a Monte Carlo simulation is performed, slightly different results (mean values) are obtained. The variability of results (i.e., how much the mean estimate varies from one Monte Carlo simulation to another Monte Carlo simulation) depends on the number *N* of trials in each Monte Carlo simulation.

Let *x*_*i*_, *i* ∈ {1, …, *N*} denote the individual randomly generated values, let *N* be the sample size, let $\bar{x}$ denote the mean value of the sample and let $\sigma_{x}^{2}$ be its variance. When Monte Carlo simulations are repeated, the mean values $\bar{x}$ will slightly differ (variances are assumed to be identical). The variance of the mean $\sigma_{\bar{x}}^{2}$ is then given as follows [41]:

$$\begin{array}{c} \sigma_{\bar{x}}^{2}=\frac{\sigma_{x}^{2}}{N} \end{array}$$ (1)

Thus, the standard error (deviation) of the mean $\sigma_{\bar{x}}$ decreases with the square root of the sample size *N* in each Monte Carlo simulation. This relation does not depend on the underlying probability distribution.

In our approach, the criteria weights are randomly drawn from the uniform probability distribution. For the uniform probability distribution, where *x*_*i*_ ∈ [*a*, *b*], the variance $\sigma_{x}^{2}$ is given as follows:

$$\begin{array}{c} \sigma_{x}^{2}=\frac{{\left(b-a\right)}^{2}}{12} \end{array}$$ (2)

The relations above enable estimation of the sample size *N* so that a standard error is under a desired threshold, see Section 4 for more details.

### 2.2 Monte Carlo weights and dominance relations

Let *A* = {*A*_1_, *A*_2_, …, *A*_*k*_} be the set of *k* alternatives under consideration, let *C* = {*C*_1_, *C*_2_, …, *C*_*n*_} be the set of *n* criteria and let *w* = (*w*_1_, *w*_2_, …, *w*_*n*_) be the vector of criteria weights such that *w*_*i*_ ∈ ]*a*, *b*[, *b* > *a* > 0.

Since in our approach the weights of criteria are randomly generated from the interval [*a*, *b*[ by the Monte Carlo method, we will denote these weights as *Monte Carlo weights* (*MC weights* in short). For practical purposes the number of generated cases of these weights is recommended to be at least in thousands, see e.g. [42, 43], or [44], but see Section 4 for more details.

Further on, let’s assume that all alternatives are evaluated with respect to all criteria, and *f*_*ij*_ denotes the evaluation of the *i*-th alternative under *j*-th criterion, where *f*_*ij*_ ∈ *R*. The matrix *F* = (*f*_*ij*_) is called the *decision matrix*. Further on, let *U*(*A*_*i*_) be a (cardinal) utility function of an alternative *i*:

$$\begin{array}{c} U\left(A_{i}\right)=\sum_{j=1}^{n}f_{ij}\cdot w_{j}. \end{array}$$ (3)

Next, we propose the following dominance relations for alternatives’ comparison and ranking.

**Definition 1**

*Let N be the number of cases of Monte Carlo weights w = (w*_1_, *w*_2_, …, *w*_*n*_). *Let B_i_*, ∀*i* ∈ {1, …, *n*}, *be the number of generated MC weights such that alternative A_i_ achieved the highest value of* Eq (3) (*was the best*) *from the set of all alternatives. Then A_i_ dominates A_j_ (A_i_* ≻ *A_j_) w. r. t. Definition 1 if and only if B_i_* > *B_j_*.

**Definition 2**

*Let N be the number of cases of Monte Carlo weights w = (w*_1_, *w*_2_, …, *w*_*n*_). *Let D_ij_*, ∀*i* ∈ {1, …, *n*}, *be the number of generated cases such that alternative A_i_ achieved a higher value of the utility function* (3) *than alternative A_j_. Then A_i_ dominates A_j_ (A_i_* ≻ *A_j_) w. r. t. Definition 2 if and only if D_ij_* > *D_ji_*.

**Definition 3**

*Let N be the number of cases of Monte Carlo weights w = (w*_1_, *w*_2_, …, *w*_*n*_). *Let* $U_{mean}^{i}=\frac{1}{N}\sum_{r=1}^{N}\sum_{j=1}^{n}f_{i}^{\left(j\right)}\cdot w^{\left(j\right)}$ *be the mean utility function achieved by alternative A_i_ over all generated cases. Then alternative A_i_ dominates A_j_ (A_i_* ≻ *A_j_*) *w. r. t. Definition 3 if and only if* $U_{mean}^{i}>U_{mean}^{j}$.

By each of the three dominance relations alternatives can be partially ordered (in the case of ties) or totally ordered (in the case of no ties).

**Remark 1**

*Let T be the number of ties where U_i_ = U_j_ out of N generated cases. A matrix D = (d_ij_) such that d_ij_ = D_ij_/(N–T) forms a square pairwise comparison matrix denoted as a* ‘*fuzzy*’ *PC matrix with elements satisfying the relation d_ij_ + d_ji_* = 1, *which, in turn, can be easily transformed into a multiplicative PC matrix A* = (*a*_*ij*_) *via relation* $a_{ij}=\frac{d_{ij}}{1-d_{ij}}$. From a multiplicative PC matrix alternatives’ weights (also called a *priority vector*) can be easily derived by the eigenvalue method or the geometric mean method, see e.g. [45, 46], or [39].

Further on, we define *central weights* (the most stable weights) for each alternative as follows:

**Definition 4**

*Let f*_*ij*_ *be the evaluation of alternative i with respect to criterion j. Let w* = (*w*_1_, …, *w*_*n*_) *be a vector of MC weights of all criteria. Let U^(i)^ denote a utility function of alternative i. Let W*^(*i*)^ = {*w*|*U*^*i*^ ≥ *U*^*j*^, ∀*j*} *be a “space” of weights for which alternative i is the best (attains the maximum value of a utility function). Further on, let* $w_{*}^{\left(i\right)}\in W^{\left(i\right)}$ *denote weights for which two conditions are satisfied*:

*i) There exists a neighbourhood in the form of an open “ball” N* ⊂ *W*^(*i*)^ *such that* $w_{*}^{\left(i\right)}$ *is its centre, and r* > 0 *is its radius*.

*ii) The radius r is maximal*.

*Then the* $w_{*}^{\left(i\right)}$ *is called the central weights w.r.t. alternative i*.

Obviously, the greater is the value of *r* from Definition 4, the greater is the necessary change in weights from central weights to replace the best alternative *i* with another best alternative. In this sense, *r* expresses stability or robustness of the central weights. Also, it should be mentioned that the previous definition utilizes the notion of a distance (between weights), hence a suitable metric function must be selected in practice. Therein after, it is assumed that the *Manhattan metric* is such a suitable metric, see also [47].

The best or optimal alternative in MCDM/MCDA problems always belongs to the set of non-dominated alternatives. This means that given the set of alternatives *A* = {*A*_1_, …, *A*_*k*_} and the set of criteria *C* = {*C*_1_, …, *C*_*n*_}, alternative *A*_*i*_ dominates alternative *A*_*j*_ (we write *A*_*i*_ ≻ *A*_*j*_) if for all *j* = {1, …, *n*} it holds that *A*_*i*_ is evaluated better or equally as *A*_*j*_, but at least one preference is strict.

Next, we provide a generalization of the concept of dominance.

**Definition 5**

*Let A = {A*_1_, …, *A*_*k*_} *be the set of alternatives and let C* = {*C*_1_, …, *C*_*n*_} *be the set of criteria. Let N* ≥ *N*_0_ be the number of randomly generated MC weights. We say that alternatives from the set A* ⊆ *A dominate alternative A_j_ w. r. t*. *Definition 5, if for each generated case of MC weights there is an alternative A_i_* ∈ A* *such that A*_*i*_ ≻ *A*_*j*_.

In other words, if an alternative *j* is dominated by a set of alternatives according to Definition 5, it is never ranked as the best one. We recommend to set the lower bound *N*_0_ of the number of randomly generated MC weights to 10,000 in accordance with [42, 43], or [44].

While the case of one alternative dominance over another alternative can be called *single-dominance* (*s-dominance* in short), the case of the dominance of a set over one alternative can be referred to as *multi-dominance* (*m-dominance* in short). It should be noted that while *s-dominance* implies *m-dominance*, the inverse is not true in general.

To summarize, the proposed Monte Carlo weights method for multiple criteria decision analysis proceeds in the following steps (that slightly differ with regard to the dominance relation involved):

1) The sets of alternatives *A* and criteria *C* along with the decision matrix *F* = (*f*_*ij*_) form the method’s input. Also, a probability distribution of random draws of weights is set (usually it is uniform distribution).

2) A large number of criteria weights is generated randomly via Monte Carlo method such that each weight *w*_*i*_ is drawn independently from (the same) open interval ]*a*, *b*[. The dominance relation is selected.

3i) For the dominance relation from Definition 1: For each generated case of the MC weights the best alternative (the alternative with the highest value of a utility function) is found.

4i) Results are aggregated over all generated cases and the values of *B*_*i*_ are found.

5i) All alternatives are ranked via the dominance relation from Definition 1 from the best to the worst.

3ii) For the dominance relation from Definition 2: For each generated case of the MC weights all alternatives are pairwise compared with respect to their utility function.

4ii) Results are aggregated over all generated cases and the values of *D*_*ij*_ are found.

5ii) All alternatives are ranked via the dominance relation from Definition 2 from the best to the worst.

3iii) For the dominance relation from Definition 3: For each generated case of the MC weights the value of a utility function is calculated for each alternative.

4iii) Results are aggregated over all generated cases and the values of $U_{mean}^{i}$ are found.

5iii) All alternatives are ranked via the dominance relation from Definition 3 from the best to the worst.

6) At this final step central weights w_*_ and radius *r* are estimated for each alternative.

**Fig 1:**
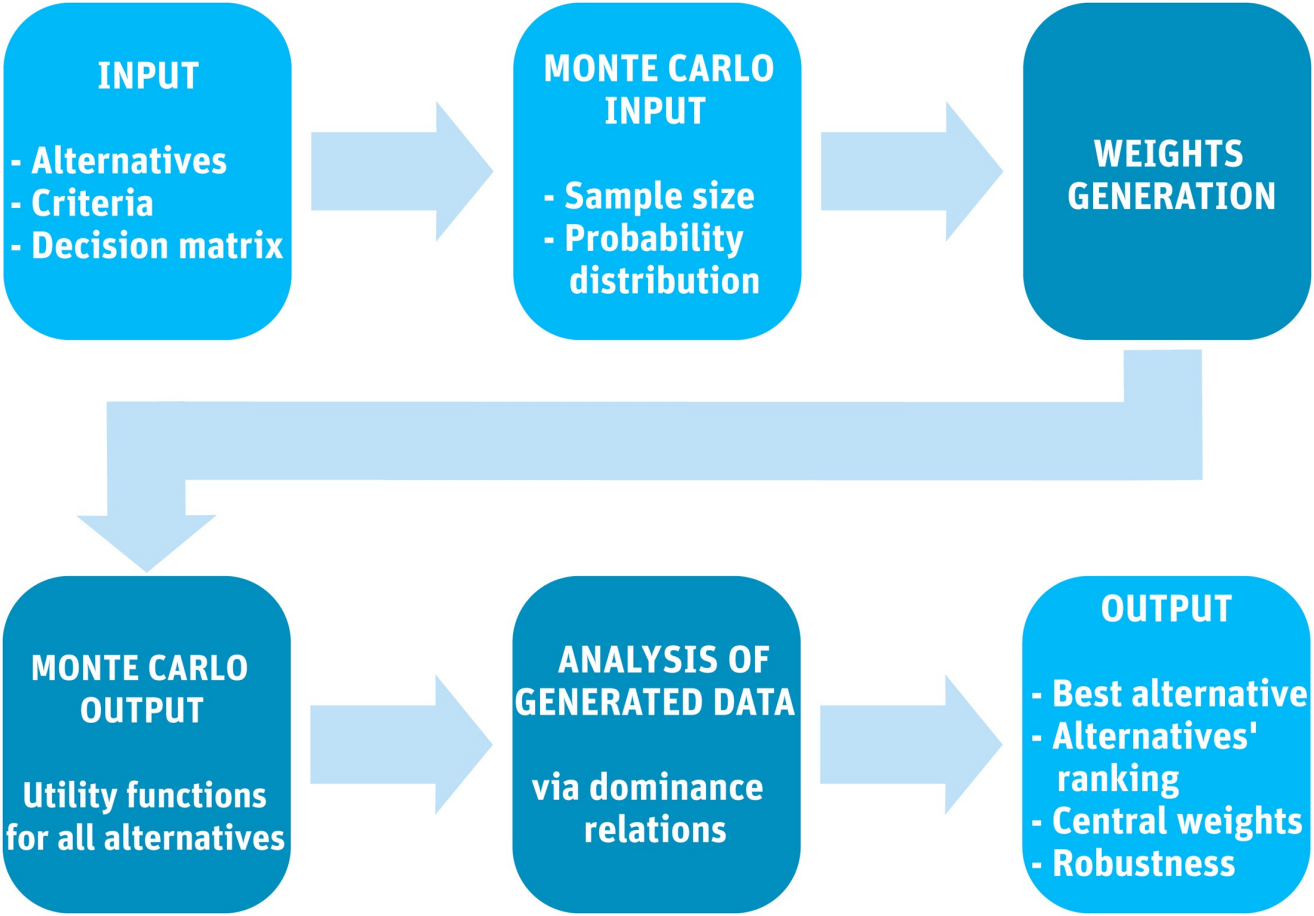
Monte Carlo weights method.

Fig 1 shows a simplified flow chart of the method. During the procedures above a decision maker may identify an alternative that is multi-dominated, i.e. never best and thus irrelevant. In such a case it is recommended to remove this alternative from further consideration.

### 2.3 An illustrative numerical example

Let’s consider 5 alternatives {*A*_1_, *A*_2_, *A*_3_, *A*_4_, *A*_5_}, and 3 criteria {*C*_1_, *C*_2_, *C*_3_}. All alternatives are evaluated on the scale from 1 (the worst) to 10 (the best), see Table 1. Weights of all three criteria are unknown. The goal is to find the best alternative.

**Table 1:** The decision matrix: The evaluation of alternatives with respect to criteria.

Alternative/criteria	*C*_1_	*C*_2_	*C*_3_
*A*_1_	7	7	4
*A*_2_	2	8	5
*A*_3_	3	4	5
*A*_4_	1	8	8
*A*_5_	2	2	10

To solve the problem we use the Monte Carlo weights method with 10,000 randomly generated criteria weights. For each generated case, the dominance relations from Definitions 1–3 were applied and the results are presented in Tables 2–5. We use the same number of generated cases (a sample size) through the paper purely for practical reasons—we built our Monte Carlo simulation tool with 10,000 cases. This simple size is usually more than sufficient and provides robust results, however, a researcher may adjust the sample size with respect to the desired accuracy, see Discussion (Section 4) for more details.

**Table 2:** Alternatives’ evaluation with respect to Definitions 1 and 3.

Alternative	Best w.r.t. Def. 1 (in %)	*U*_*mean*_
*A*_1_	34.9	8.02
*A*_2_	0.67	7.52
*A*_3_	0	6.02
*A*_4_	45.7	8.52
*A*_5_	18.7	7.03

**Table 3:** Alternatives’ evaluation with respect to Definition 2.

Alternative	*A*_1_	*A*_2_	*A*_3_	*A*_4_	*A*_5_
*A*_1_	–	6692	9731	3894	6461
*A*_2_	3304	–	8741	1714	5791
*A*_3_	269	1258	–	583	3005
*A*_4_	6104	8283	9416	–	7485
*A*_5_	3538	4209	6992	2514	–

**Table 4:** Alternatives’ evaluation with respect to Definition 2 and Remark 1, a fuzzy preference matrix.

Alternative	*A*_1_	*A*_2_	*A*_3_	*A*_4_	*A*_5_
*A*_1_	0.5	0.669	0.973	0.389	0.646
*A*_2_	0.330	0.5	0.874	0.171	0.579
*A*_3_	0.027	0.126	0.5	0.058	0.301
*A*_4_	0.610	0.828	0.942	0.5	0.749
*A*_5_	0.354	0.421	0.699	0.251	0.5

**Table 5:** Alternatives’ evaluation with respect to Definition 2 and Remark 1, a multiplicative preference matrix.

Alternative	*A*_1_	*A*_2_	*A*_3_	*A*_4_	*A*_5_
*A*_1_	1	2.02	36.17	0.64	1.83
*A*_2_	0.49	1	6.94	0.21	1.38
*A*_3_	0.027	0.14	1	0.06	0.43
*A*_4_	1.57	4.82	16.12	1	2.98
*A*_5_	0.55	0.73	2.32	0.34	1

**Fig 2:**
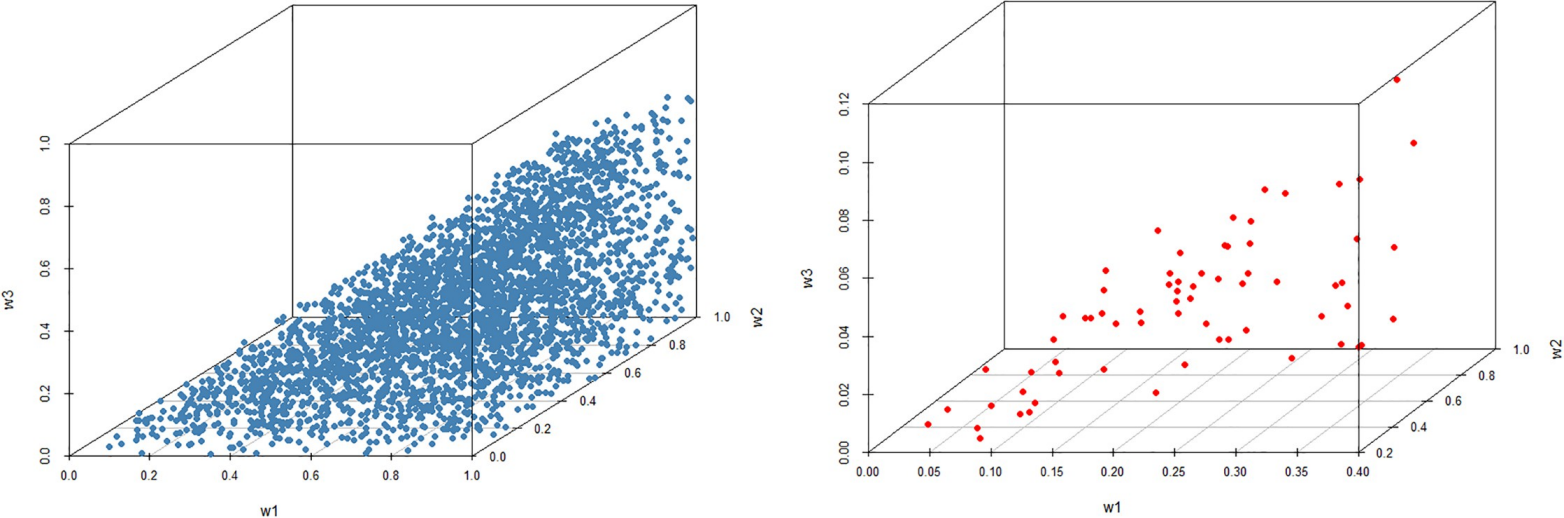
Weights of criteria if A1 is the best alternative (left) and A2 is the best alternative (right).

**Fig 3:**
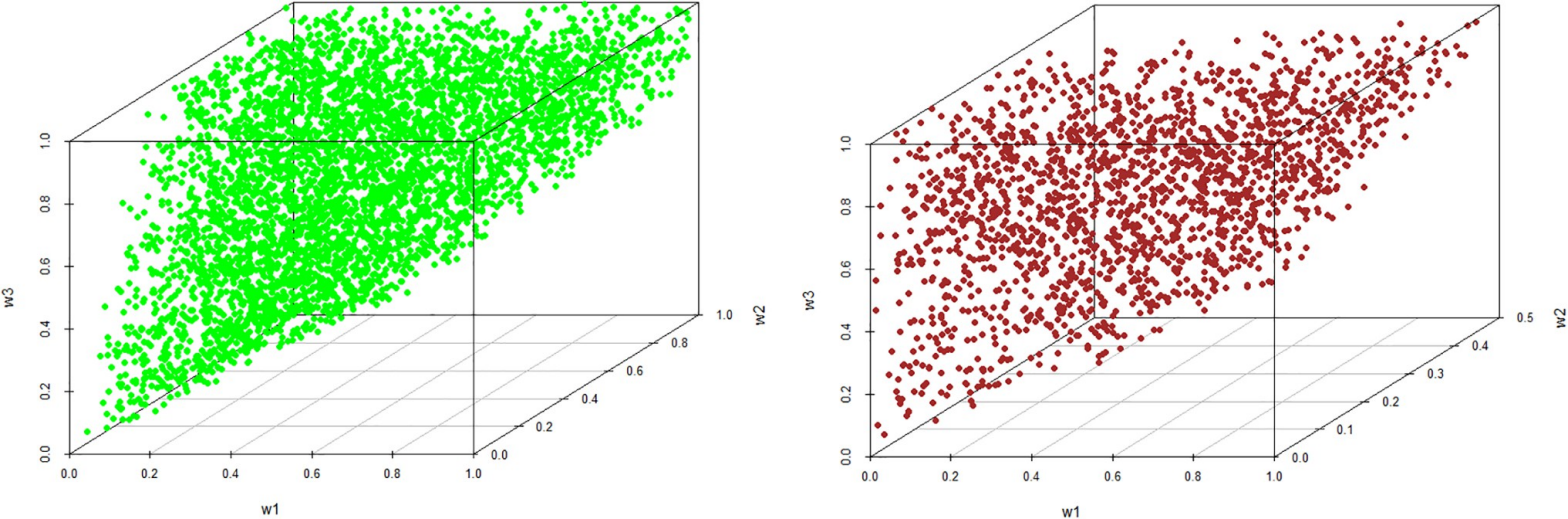
Weights of criteria if A4 is the best alternative (left) and A5 is the best alternative (right).

As can be seen, the best alternative (a Condorcet winner) is *A*_4_ followed by *A*_1_. Figs 2 and 3 illustrate a ‘space’ of weights for which a given alternative is ranked best. Rankings of all alternatives with respect to dominance relations from Definitions 1–3 are provided in Table 6. The weights of all alternatives from the PC matrix in Table 5 derived by the geometric mean method are as follows: *w*_*GM*_ = (0.319, 0.130, 0.021, 0.426, 0.104).

**Table 6:** Alternatives’ rankings with respect to Definitions 1–3.

Rank	Def. 1	Def. 2	Def. 3
1	*A*_4_	*A*_4_	*A*_4_
2	*A*_1_	*A*_1_	*A*_1_
3	*A*_5_	*A*_2_	*A*_2_
4	*A*_2_	*A*_5_	*A*_5_
5	*A*_3_	*A*_3_	*A*_3_

**Fig 4:**
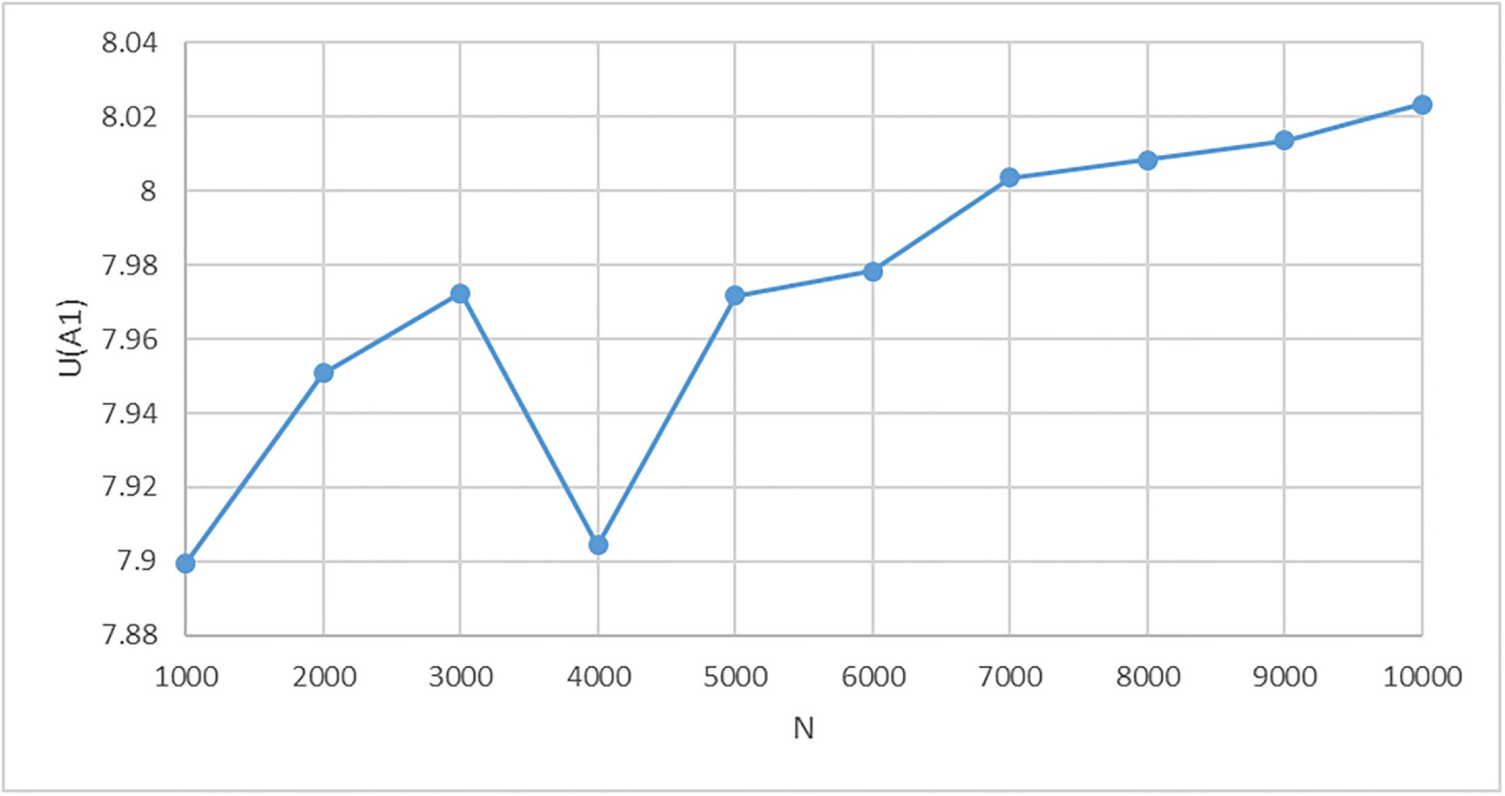
The dependence of *U*(*A*_1_) mean values on *N*.

A natural question regarding this or other problems associated with the Monte Carlo method arises: how many cases should be randomly generated so that the result is robust enough. We provide an answer to this question in Section 4. Here, we show the convergence of *U*_*mean*_(*A*_1_) with the growing number *N* of generated cases, see Fig 4. For *N* = 1, 000 the value of *U*_*mean*_(*A*_1_) = 7.90, for *N* = 2, 000 is *U*_*mean*_(*A*_1_) = 7.95, and for *N* = 5, 000 is *U*_*mean*_(*A*_1_) = 7.97. These values differ from the value 8.024 (*N* = 10, 000) by 1.5%, 0.9% and 0.6% respectively (this deviation should not be confused with the *relative standard error* introduced in Section 4), hence, even with *N* = 2, 000 the deviation of the value of the utility function *U*_*mean*_(*A*_1_) from the value for *N* = 10, 000 is under 1%.

## 3 Application of Monte Carlo weights to the wind turbine selection

In the study of Rehman and Khan [48], the task of finding the best wind turbine for a wind power plant was performed. The authors gathered data about 18 wind turbines and evaluated their properties with regard to five criteria: hub height (*C*_1_), rotor diameter (*C*_2_), cut-in speed of wind (*C*_3_), rated speed of wind (*C*_4_) and rated power (*C*_5_). Every criterion had the weight equal to 0.20. The data were normalized, criteria *C*_1_ − *C*_4_ were minimization ones, criterion *C*_5_ was originally a maximization one, so it was transformed into a minimization one by taking its inverse. After the transformation all criteria were minimization ones and the best turbine was the turbine with the lowest weighted sum—Fuhrlander FL 600, see Table 7.

**Table 7:** The evaluation of all turbines with respect to all criteria, normalized matrix, [48].

Turbine	Normalized Hub Height	Normalized Rotor Diameter	Normalized Cut-in Speed of wind	Normalized Rated Speed of wind	Normalized Rated Power	Weighted Sum
Fuhrlander FL 600	0.588	0.526	0.357	0.688	0.714	0.575
Hyosung HS50	0.588	0.526	0.500	0.688	0.643	0.589
RRB Energy PS 600	0.565	0.495	0.500	0.938	0.714	0.642
Suzlon S.52/600	0.882	0.547	0.571	0.813	0.714	0.706
Unison U57	0.800	0.600	0.429	0.656	0.643	0.626
Vestas V47	0.647	0.495	0.571	0.813	0.686	0.642
Windflow 500	0.341	0.347	0.857	0.875	0.762	0.637
AAER A-1000	0.824	0.611	0.571	0.750	0.524	0.656
DeWind D6 64m	0.706	0.674	0.357	0.769	0.405	0.582
Mitsubishi MWT62	0.812	0.646	0.500	0.781	0.524	0.653
Nordex N54/1000	0.706	0.568	0.536	0.875	0.524	0.642
Suzlon S.62/1000	0.765	0.653	0.429	0.750	0.524	0.624
Vensys 62-1200	0.812	0.653	0.357	0.719	0.429	0.594
AAER A-2000-84	0.765	0.884	0.464	0.750	0.048	0.582
DeWind D.81	0.941	0.842	0.429	0.848	0.048	0.621
Ecotecnia 80/2000	0.824	0.842	0.429	0.750	0.048	0.578
REpower MM92	0.929	0.968	0.429	0.781	0.048	0.631
Suzlon S.88/2000	0.941	0.926	0.571	0.875	0.048	0.672

What escaped notice of the authors of the study is that Suzlon S.52 and Suzlon S.88 turbines were dominated by other alternatives, so they could be safely removed from further consideration.

As can be seen from Table 7, differences between turbines’ final scores were rather small. Therefore, it could be expected that even a small change in criteria weights might lead to a different best wind turbine. Indeed, it suffices to change the weight of the criterion *C*_2_ to 0.195 and the weight of the criterion *C*_5_ to 0.205 (leaving the rest of criteria weights at 0.20), and the weighted sum for Fuhrlander FL 600 changes to 0.5755, while Ecotecnica 80/2000 attains 0.5746, becoming the best one. It took only 1% change of weights to arrive at a different best alternative.

To analyse the dependence of the best turbine on criteria weights, we applied the Monte Carlo weights method. We generated 10,000 random cases of criteria weights from the interval ]0, 1[ and applied the dominance relations from Definition 1 and 2 to compare and rank all turbines (except for the two dominated ones) and to find their respective central weights—this dataset can be found on https://doi.org/10.6084/m9.figshare.19525087.v1. The results are summarized in Tables 8 and 9.

**Table 8:** Turbines, their central weights w*, radius *r* and a percentage of cases in which they were ranked first.

Turbine	*w*_1_	*w*_2_	*w*_3_	*w*_4_	*w*_5_	*r*	Best (in %)	mean *U*
Fuhrlander FL 600	0.526	0.552	0.636	0.553	0.275	0.498	36.2	1.4404
Hyosung HS50	0.329	0.595	0.114	0.781	0.384	0.277	1.2	1.4768
RRB Energy PS 600	–	–	–	–	–	–	0	1.6103
Suzlon S.52/600	–	–	–	–	–	–	0	1.7691
Unison U57	0.022	0.075	0.168	0.704	0.086	0	0.04	1.5691
Vestas V47	–	–	–	–	–	–	0	1.6106
Windflow 500	0.690	0.657	0.192	0.389	0.349	0.371	15.3	1.5945
AAER A-1000	–	–	–	–	–	–	0	1.6458
DeWind D6 64m	0.378	0.483	0.819	0.132	0.461	0.247	1.1	1.4609
Mitsubishi MWT62	–	–	–	–	–	–	0	1.6373
Nordex N54/1000	–	–	–	–	–	–	0	1.6098
Suzlon S.62/1000	–	–	–	–	–	–	0	1.5659
Vensys 62-1200	0.063	0.610	0.713	0.488	0.429	0.202	0.8	1.4906
AAER A-2000-84	0.686	0.250	0.392	0.507	0.726	0.463	16.3	1.4624
DeWind D8.1	–	–	–	–	–	–	0	1.5595
Ecotecnia 80/2000	0.300	0.495	0.556	0.494	0.735	0.478	29.1	1.4535
REpower MM92	–	–	–	–	–	–	0	1.5853
Suzlon S.88/2000	–	–	–	–	–	–	0	1.6885

**Table 9:** Turbines and their dominance w.r.t. Definition 2.

	Fuhrl- ander FL 600	Hyosung HS50	RRB Energy PS 600	Unison U57	Vestas V47	Windflow 500	AAER A-1000	DeWind D6 64m	Mitsubi- shi MWT62	Nordex N54/1000	Suzlon S.62/1000	Vensys 62-1200	AAER A-2000-84	DeWind D8.1	Ecotecnia 80/2000	REpower MM92
Fuhrlan- der FL 600	–	7551	9875	9687	9949	7866	9684	5635	9716	9554	9189	6630	5344	6767	5190	7046
Hyosung HS50	2449	–	9772	9032	9994	7718	9829	4408	9726	9627	8734	5503	4777	6323	4654	6707
RRB Energy PS 600	125	228	–	3702	5057	4525	6157	1274	5958	4963	3456	2038	2846	4281	2726	4692
Unison U57	313	968	6298	–	6848	5437	8909	1157	8830	6743	4756	1472	3221	4811	3081	5259
Vestas V47	51	6	4943	3152	–	4514	6651	1052	6229	4948	3081	1660	2701	4247	2596	4645
Windflow 500	2134	2282	5475	4563	5486	–	6008	2876	5833	5340	4470	3443	3393	4594	3325	4895
AAER A-1000	316	171	3843	1091	3349	3992	–	60	3787	2553	344	26	1436	3226	1245	3774
DeWind D6 64m	4365	5592	8726	8843	8948	7124	9940	–	9998	9786	9967	7745	5070	7512	4813	7870
Mitsubi- shi MWT62	284	274	4042	1170	3771	4167	6213	2	–	2878	4	1	1447	3392	1239	3965
Nordex N54/1000	446	373	5037	3257	5052	4660	7447	214	7122	–	2293	795	2111	4016	1936	4548
Suzlon S.62/1000	811	1266	6544	5244	6919	5530	9656	33	9996	7707	–	218	2826	4845	2651	5393
Vensys 62-1200	3370	4497	7962	8528	8340	6557	9974	2255	9999	9205	9782	–	4280	6808	4064	7291
AAER A-2000-84	4656	5223	7154	6779	7299	6607	8564	4930	8553	7889	7174	5720	–	9478	3586	9952
DeWind D8.1	3233	3677	5719	5189	5753	5406	6774	2488	6608	5984	5155	3192	522	–	0	7034
Ecotecnia 80/2000	4810	5346	7274	6919	7404	6675	8755	5187	8761	8064	7349	5936	6414	10000	–	9999
REpower MM92	2954	3293	5308	4741	5355	5105	6226	2130	6035	5452	4607	2709	48	2966	1	–

The turbine that was the most frequently best (in 36.2% of generated cases) was Fuhrlander FL 600 in accord with the result of the original study. However, our approach provided new valuable insights into the problem. Firstly, it can be seen from the Table 7 that another 8 turbines were *m*-dominated and would never be ranked the best (with both Suzlon turbines mentioned above 10 turbines altogether attained 0% cases of being evaluated as the best). From the remaining 8 turbines only four turbines were ranked best in at least 10% of cases: Fuhrlander FL 600, Windflow 500, AAER A-2000-84 and Ecotecnia 80/2000. Only these four turbines could deserve a more detailed consideration. Therefore, the Monte Carlo approach allowed significant reduction of the candidates for the best solution. But the advantages of the method do not stop here.

From the central weights for each of the four best alternatives, we see what weights would favour one alternative over the others. Windflow 500 turbine would be considered the best if criteria *C*_1_ and *C*_2_ were the most important. AAER A-2000-84 turbine would be best if criteria *C*_1_ and *C*_5_ were the most important, and finally Ecotecnia 80/2000 would be ranked first if the last criterion *C*_5_ was the most important and the first criterion *C*_1_ was the least important. After this analysis a decision maker may weigh in which configuration of criteria weights is most desirable, and then select the best option.

## 4 Discussion

The Monte Carlo weights method for the solution of MCDM/MCDA problems involving a utility function has several advantages, namely:

- The method does not need precise values of criteria weights in advance since it models a large number of feasible weights so that the decision maker receives information of how the weights influence the results.
- The method enables the evaluation and ranking of alternatives despite the unknown criteria weights.
- The method enables to find the set of weights for which a given alternative is the best. Then it is up to the decision maker to decide which weights are acceptable and which are not.
- The method enables the evaluation of a stability of the so called central weights. The concept of central weights enables the decision maker to see what weights would be necessary for each alternative to be the best one.
- The method enables, as shown in the example on the wind turbine selection, to find multi-dominated alternatives, which are not so obvious as their single-dominated counterparts, thus reducing the number of alternatives under consideration.

On the other hand, the Monte Carlo weights approach has its limitations. Firstly, in some real-world problems criteria weights are set a priori at given values and the analysis of what would happen if they change is irrelevant. Secondly, Monte Carlo method is both computationally costly and time demanding, and might not be useful in situations when a fast solution is needed. Other limitation constitutes the fact that we introduce Monte Carlo weights for the problems where the final aggregation of alternatives’ evaluations is performed via a utility function, but many MCDM/MCDA theoretical frameworks do not incorporate a utility function. However, we believe the Monte Carlo weights can be introduced into other frameworks associated with criteria weights as well, and our future research will focus in this direction.

As mentioned in Section 2, it is useful to know the size of the randomly generated sample necessary for results to be robust enough. Hereinafter, we provide this estimate.

First we assume the criteria weights are randomly drawn from a uniform distribution on the interval ]*a*, *b*[. Let *x*_*i*_ denote randomly generated values of weights of a given criterion (does not matter which one, since they are treated equally), let $\bar{x}=(b-a)/2$ denote the mean weight of a given criterion (this value is the same for all criteria), let $\sigma_{x}^{2}=\frac{(b-a{)}^{2}}{12}$ be the sample variance of *x*_*i*_ and let $\sigma_{\bar{x}}^{2}$ be the standard error (variance) of the mean. From relations (1) and (2) it follows that

$$\begin{array}{c} \sigma_{\bar{x}}^{2}=\frac{{\left(b-a\right)}^{2}}{12N}. \end{array}$$ (4)

Further on, let $\frac{\sigma_{\bar{x}}}{\bar{x}}=p$, where *p* is *the coefficient of variation of the mean* (also called *the standard error of the mean* or *the relative standard error*).

Now, let’s estimate the sample size *N* corresponding to the relative standard error *p*:

$$\begin{array}{c} \frac{\sigma_{\bar{x}}}{\bar{x}}=p=\frac{\frac{\left(b-a\right)}{\sqrt{12N}}}{\frac{\left(b-a\right)}{2}}=\frac{2}{\sqrt{12N}} \end{array}$$ (5)

hence

$$\begin{array}{c} N=\frac{1}{3p^{2}}. \end{array}$$ (6)

The relation (6) provides the relationship between the sample size *N* and the relative standard error *p* (given as a decimal number) of a given generated weight. The smaller is *p*, the more ‘fairly’ are the weights generated (no weight is, on average, higher than some other weight), however, the price is a large sample *N*.

For reader’s convenience, we provide the sample sizes *N* for different values of *p* in the following Table 10.

**Table 10:** The (minimal) sample size *N* with respect to the relative standard error *p*.

*p*	0.01	0.02	0.03	0.04	0.05	0.10
N	3,333	833	370	208	133	33

It should be noted that the sample sizes *N* provided by relation (6) and shown in Table 10 are only estimates since a sample variance of *x*_*i*_ is used instead of (unknown) population variance, and the assumption of (totally) random draws might not be fulfilled in practice due to the application of pseudo-random generators mentioned in Section 2.1.

An estimate of the sample size with respect to the relative standard error of a utility function *U* can be derived as well. Assume the utility function from relation (3) and recall the following formula for the variance of a linear combination of two uncorrelated (independent) variables (*x*, *y*):

$$\begin{array}{c} \sigma^{2}\left(ax+by\right)=a^{2}\sigma^{2}\left(x\right)+b^{2}\sigma^{2}\left(y\right) \end{array}$$ (7)

Now, let’s estimate the relative standard error of the mean of the utility function of an alternative *j* (we assume *f*_*ij*_ ≥ 0). The variance of *U*_*j*_ is given as:

$$\begin{array}{c} \sigma_{U_{j}}^{2}=\sigma^{2}\left(\sum_{i}^{n}f_{ij}w_{i}\right)=f_{1j}^{2}\cdot \sigma_{w_{1}}^{2}+\ldots +f_{nj}^{2}\cdot \sigma_{w_{n}}^{2}=\frac{{\left(b-a\right)}^{2}}{12}\sum_{i}f_{ij}^{2} \end{array}$$ (8)

Therefore, the variance of the mean of *U*_*j*_ is given as follows:

$$\begin{array}{c} \sigma_{\bar{U_{j}}}^{2}=\frac{\frac{{\left(b-a\right)}^{2}}{12}\sum_{i}^{n}f_{ij}^{2}}{N} \end{array}$$ (9)

And the relative standard error *p* is given as:

$$\begin{array}{cc} p=\frac{\sigma_{\bar{U_{j}}}}{\bar{U_{j}}}= & \frac{\sqrt{\sum_{i}f_{ij}^{2}}\left(b-a\right)}{\sqrt{12N}\bar{U_{j}}}=\frac{\sqrt{\sum_{i}f_{ij}^{2}}\left(b-a\right)}{\sqrt{12N}\sum_{i}f_{ij}\bar{w_{i}}}\\ = & \frac{2\sqrt{\sum_{i}f_{ij}^{2}}\left(b-a\right)}{\sqrt{12N}\left(b-a\right)\sum_{i}f_{ij}}=\frac{\sqrt{\sum_{i}f_{ij}^{2}}}{\sqrt{3N}\sum_{i}f_{ij}} \end{array}$$ (10)

Finally, from Eq (10) we easily derive *N*:

$$\begin{array}{c} N=\frac{\sum_{i}f_{ij}^{2}}{3p^{2}{\left(\sum_{i}f_{ij}\right)}^{2}} \end{array}$$ (11)

Since for *f*_*ij*_ ≥ 0 the following inequality holds:

$$\begin{array}{c} \sum_{i}f_{ij}^{2}\leq {\left(\sum_{i}f_{ij}\right)}^{2}. \end{array}$$ (12)

We get that the following estimate:

$$\begin{array}{c} N\leq \frac{1}{3p^{2}}. \end{array}$$ (13)

The sample size estimate for the relative standard error of the mean of a utility function is thus lower than the sample size estimate for the relative standard error of the mean of a given weight.

Another interesting problem is whether it is possible to obtain a set of weights *W* for which a given alternative is the best (has the highest value of the utility function *U*), see Definition 1, analytically, without simulations. Assume that there are at least two alternatives and that weights of criteria *w*_*i*_ ∈ ]0, 1[.

This task means to solve a system of linear inequalities where the number of inequalities equals the number of alternatives (*k*) minus one plus 2*n* ‘structural inequalities’ (0 < *w*_*i*_ < 1), and the number of variables is equal to the number of criteria (*n*). Suppose that we want to find the set *W*_1_ for Alternative 1. Then, the system of inequalities is given as follows:

$$\begin{array}{c} \left\{ \begin{array}{c} f_{11}w_{1}+f_{12}w_{2}+\ldots +f_{1n}w_{n}\geq f_{21}w_{1}+f_{22}w_{2}+\cdots +f_{2n}w_{n}\\ \ldots \\ f_{11}w_{1}+f_{12}w_{2}+\cdots +f_{1n}w_{n}\geq f_{k1}w_{1}+f_{k2}w_{2}+\ldots +f_{kn}w_{n}\\ 0<w_{i}<1,\forall i. \end{array}\;.\right. \end{array}$$ (14)

This set of inequalities can be solved by the *Fourier–Motzkin elimination (FME)*, see e.g. [49]. In each step of the FME, one variable is eliminated from the system, but new inequalities are added, until only one variable remains and its value can be expressed as an interval. Let’s assume that the solution to the system above was found and has the following form, where *L* and *U* denote the lower and upper bounds for each weight *w*_*i*_:

$$\begin{array}{c} \left\{ \begin{array}{c} L_{1}\left(w_{2},\ldots ,w_{n}\right)\leq w_{1}\leq U_{1}\left(w_{2},\ldots ,w_{n}\right)\\ L_{2}\left(w_{3},\ldots ,w_{n}\right)\leq w_{2}\leq U_{2}\left(w_{3},\ldots ,w_{n}\right)\\ \ldots \\ L_{n}\leq w_{n}\leq U_{n} \end{array}\;.\right. \end{array}$$ (15)

The set *W*_1_ forms a polyhedron in an *n*-dimensional unit cube. For the comparisons with other alternatives with regard to Definition 1, instead of counting the number of generated cases for which Alternative 1 is the best, we have to find the volume of the set *W*_1_. This volume is given as an *n*-dimensional definite integral:

$$\begin{array}{c} vol\left(W_{1}\right)=\int_{L_{n}}^{U_{n}}\int_{L_{n-1}\left(w_{n}\right)}^{U_{n-1}\left(w_{n}\right)}\ldots \int_{L_{1}\left(w_{2},\ldots ,w_{n}\right)}^{U_{1}\left(w_{2},\ldots ,w_{n}\right)}1dw_{1}dw_{2}\ldots dw_{n}. \end{array}$$ (16)

However, the downside of the FME is that the number of inequalities grows doubly exponentially [50, 51]. At most, one can expect to get $4(\frac{k}{4}{)}^{2^{n-1}}$ inequalities for one variable, where *k* is the input number of inequalities and *n* is the number of variables [51]. Hence, for instance, with originally 8 inequalities and 4 variables one may end up (in the worst case scenario) with a system of 1,024 inequalities that leads to the solution for only one alternative… Therefore, it is possible to use the analytic approach, but the computational complexity makes it rather infeasible in practice except for the cases with very low numbers of alternatives and criteria.

## 5 Conclusions

In this paper we introduced the notion of the Monte Carlo weights in the MCDM/MCDA framework. We showed that alternatives can be compared and ranked even when information on criteria weights is missing or unavailable, and that our approach enables to find (the most stable) weights such that a given alternative is ranked the best, and the evaluation of its stability by finding the minimal change of criteria weights necessary to a replacement at the top of the ranking. Thus, the Monte Carlo weights method provides a valuable insight into configuration of criteria weights and its influence on alternatives’ ranking.

Further on, we introduced the notion of multi-dominance, which enables to narrow the set of alternatives under consideration, and we provided estimates for Monte Carlo sample size so a desired robustness of results can be achieved.

We believe the presented approach can be useful particularly in situations when criteria weights are uncertain or difficult (impossible) to acquire, which is, in particular, the case of newly or recently emerging problems with none or insufficient previous experience.

Our further research will focus on more general framework of the Monte Carlo weights method not limited to the problems incorporating the notion of a utility function.

## References

[1] Belton V, Stewart TJ. Multiple Criteria Decision Analysis: An Integrated Approach. Kluwer: Boston; 2002.

[2] Greco S, Ehrgott M, Figueira JR. (Eds.) Multiple Criteria Decision Analysis: State of the Art Surveys. Springer-Verlag New York; 2016.

[3] Koksalan M, Wallenius J, Zionts S. Multiple Criteria Decision Making: From Early History to the 21st Century. Singapore: World Scientific; 2011.

[4] Mardani A, Jusoh A, Nor KMD, Khalifah Z, Zakwan N, Valipour A. Multiple criteria decision-making techniques and their applications—a review of the literature from 2000 to 2014. Ec Res—Ek Istr. (2015);28(1):516–571.

[5] Brugha CM. Structuring and Weighting Criteria in Multi Criteria Decision Making (MCDM). In: Stewart TJ, van den Honert RC (eds.) Trends in Multicriteria Decision Making. Lec Not in Econ & Math Syst. 1998;465. Springer, Berlin, Heidelberg.

[6] Churilov L, Flitman A. Towards fair ranking of Olympics achievements: The case of Sydney 2000. Computers and Operations Research, 2006, 33(7):2057–2082. doi: 10.1016/j.cor.2004.09.027

[7] Ginevicius R. A new determining method for the criteria weights in multicriteria evaluation. Int J Inf Technol Decis Mak. 2011;10:1067–1095. doi: 10.1142/S0219622011004713

[8] Kao C. Weight determination for consistently ranking alternatives in multiple criteria decision analysis. App Math Mod. 2010;34(7):1779–1787. doi: 10.1016/j.apm.2009.09.022

[9] Keshavarz-Ghorabaee M, Amiri M, Zavadskas EK, Turskis Z, Antucheviciene J. Determination of Objective Weights Using a New Method Based on the Removal Effects of Criteria (MEREC). Symm. 2021;13:525. doi: 10.3390/sym13040525

[10] Odu GO. Weighting methods for multi-criteria decision making technique, J App Sci & Env Man. 2019;23(8):1449–1457.

[11] Petróczy DG An alternative quality of life ranking on the basis of remittances. Socio-Economic Planning Sciences, (2021);78:101042. doi: 10.1016/j.seps.2021.101042

[12] Petróczy DG, Csató L. Revenue allocation in Formula One: A pairwise comparison approach. International Journal of General Systems, 2021, 50(3):243–261. doi: 10.1080/03081079.2020.1870224

[13] Triantaphyllou E. Multi-Criteria Decision Making: A Comparative Study. Dordrecht, The Netherlands: Kluwer Academic Publishers (now Springer); 2000.

[14] Zahir MS. Incorporating the uncertainty of decision judgements in the analytic hierarchy process European Journal of Operational Research, 1991, 53(2) 206–216. doi: 10.1016/0377-2217(91)90135-I

[15] Zardari NH, Ahmed K, Shirazi SM, Yusop ZB. Weighting Methods and their Effects on Multi-Criteria Decision Making Model Outcomes in Water Resources Management; New York, USA, Springer; 2014.

[16] Zavadskas EK, Podvezko V. Integrated determination of objective criteria weights in MCDM. Int J Inf Technol Decis Mak. 2016;15:267–283. doi: 10.1142/S0219622016500036

[17] Kaiser B. Strategy and paradoxes of Borda count in Formula 1 racing. Decyzje, 2019, 6(31):115–132.

[18] NASA Systems Engineering Handbook. 2020. https://www.nasa.gov/connect/ebooks/nasa-systems-engineering-handbook.

[19] Harrison RL. Introduction To Monte Carlo Simulation. AIP conference proceedings. 2010;1204:17–21. doi: 10.1063/1.3295638 20733932 PMC2924739

[20] Kalos MH, Whitlock PA. Monte Carlo Methods. Wiley-VCH Verlag GmbH; 2008.

[21] Kroese DP, Brereton T, Taimre T, Botev ZI. Why the Monte Carlo method is so important today. WIREs Comput Stat. 2014;6:386–392. doi: 10.1002/wics.1314

[22] Metropolis N, Ulam S. The Monte Carlo method. Am Stat Assoc. 1949;44:335–341. doi: 10.1080/01621459.1949.10483310 18139350

[23] Paxton P, Curran PJ, Bollen KA, Kirby J, Chen F. Monte Carlo Experiments: Design and Implementation. Str Eq Mod: Mult J. 2001;8(2):287–312. doi: 10.1207/S15328007SEM0802_7

[24] Eckhart R. Stan Ulam, John von Neumann, and the Monte Carlo method. Los Alamos Science (Special Issue), 1987;131–141.

[25] Doucet A, Wang X. Monte Carlo methods for signal processing: a review in the statistical signal processing context. IEEE Sig Proc Mag. 2005;22(6):152–170. doi: 10.1109/MSP.2005.1550195

[26] Kalos MH. Monte Carlo methods in the physical sciences. Proceedings of the 39th conference on Winter simulation: 40 years! The best is yet to come (WSC’07). IEEE Press. 2007;266–271.

[27] Mazurek J, Perzina R, Ramík J, Bartl D. A Numerical Comparison of the Sensitivity of the Geometric Mean Method, Eigenvalue Method, and Best–Worst Method, Math. 2021;9:554. doi: 10.3390/math9050554

[28] Seco J, Verhaegen F. Monte Carlo Techniques in Radiation Therapy (1st ed.). CRC Press; 2013.

[29] Zhu C, Liu Q. Review of Monte Carlo modeling of light transport in tissues. J Bio Opt. 2013;18(5):1–13. doi: 10.1117/1.JBO.18.5.050902 23698318

[30] Ágoston KCs, Csató L. Inconsistency thresholds for incomplete pairwise comparison matrices. Omega, 2022;108:102576. doi: 10.1016/j.omega.2021.102576

[31] Aguarón J, Moreno-Jimenez JM. The geometric consistency index: Approximated thresholds. European Journal of Operational Research, 2003, 147(1):137–145. doi: 10.1016/S0377-2217(02)00255-2

[32] Alonso JA, Lamata MT. Consistency in the analytic hierarchy process: a new approach. International Journal of Uncertainty, Fuzziness and Knowledge-Based Systems, 2006, 14(4): 445–459. doi: 10.1142/S0218488506004114

[33] Bozóki S, Rapcsák T. On Saaty’s and Koczkodaj’s inconsistencies of pairwise comparison matrices. Journal of Global Optimization, 2008, 42(2):157–175. doi: 10.1007/s10898-007-9236-z

[34] Cavallo B. Functional relations and Spearman correlation between consistency indices. Journal of the Operational Research Society, 2020, 71(2):301–311. doi: 10.1080/01605682.2018.1516178

[35] Csató L. A comparative study of scoring systems by simulations. 2021, ArXiv: 2101.05744.

[36] Csató L, Petróczy DG. On the monotonicity of the eigenvector method. European Journal of Operational Research, 2021, 292(1):230–237. doi: 10.1016/j.ejor.2020.10.020

[37] Kulakowski K, Talaga D. Inconsistency indices for incomplete pairwise comparisons matrices. International Journal of General Systems, 2020, 49(2):174–200. doi: 10.1080/03081079.2020.1713116

[38] Mazurek J, Kulakowski K. Satisfaction of the condition of order preservation. A simulation study. Operations Research and Decisions, 2020, 2: 77–89.

[39] Saaty TL. The Analytic Hierarchy Process: Planning, Priority Setting, Resource Allocation. McGraw-Hill, New York.

[40] James F, Moneta L. Review of High-Quality Random Number Generators. Computing and Software for Big Science volume. 4, 2, 2020;.

[41] Altman DG, Bland JM. Standard deviations and standard errors British Medical Journal, 331 (7521): 903. doi: 10.1136/bmj.331.7521.903 16223828 PMC1255808

[42] Burmaster DE, Anderson PD. Principles of good practice for the use of Monte Carlo techniques in human health and ecological risk assessments. Risk Analysis, 1994, 14:477–481. doi: 10.1111/j.1539-6924.1994.tb00265.x 7972955

[43] Heijungs R. On the number of Monte Carlo runs in comparative probabilistic LCA Int J Life Cycle Assess, 2020, 25: 394–402. doi: 10.1007/s11367-019-01698-4

[44] Xin L. Uncertainty and sensitivity analysis of a simplified ORWARE model for Jakarta. Stockholm, 2006. https://www.diva-portal.org/smash/get/diva2:411539/FULLTEXT01.pdf.

[45] Kulakowski K. Understanding the Analytic Hierarchy Process (1st ed.) Chapman and Hall/CRC, 2020.

[46] Ramík J. Pairwise Comparisons Method Lecture Notes in Economics and Mathematical Systems, Springer, 2020. doi: 10.1007/978-3-030-39891-0

[47] Kulakowski K, Mazurek J, Strada M. On the similarity between ranking vectors in the pairwise comparison method. J Op Res Soc. 2021.

[48] Rehman S, Khan SA. Multi-Criteria Wind Turbine Selection using Weighted Sum Approach Int J Adv Comp Sci & App. (2017);8(6):128–132.

[49] Gärtner B, Matoušek, J. Understanding and Using Linear Programming. Berlin: Springer. ISBN 3-540-30697-8; 81–104.

[50] Jing RJ, Maza MM, Talaashrafi D Complexity Estimates for Fourier-Motzkin Elimination 2019, arXiv:1811.01510v2.

[51] Lavrov M Math 482: Linear Programming, Lecture 19: Fourier–Motzkin Elimination 2019, University of Illinois at Urbana-Champaign.

